# Prevalence and Risk Factors of Chronic Kidney Disease in Patients With Type 2 Diabetes in China: Cross-Sectional Study

**DOI:** 10.2196/54429

**Published:** 2024-08-30

**Authors:** Lixin Shi, Yaoming Xue, Xuefeng Yu, Yangang Wang, Tianpei Hong, Xiaoying Li, Jianhua Ma, Dalong Zhu, Yiming Mu

**Affiliations:** 1 Department of Endocrinology and Metabolism Guiqian International General Hospital Guiyang China; 2 Department of Endocrinology and Metabolism Southern Medical University Nanfang Hospital Guangzhou China; 3 Division of Endocrinology, Department of Internal Medcine Tongji Hospital Tongji Medical College, Huazhong University of Science and Technology Wuhan China; 4 Department of Endocrinology and Metabolism The Affiliated Hospital of Medical College Qingdao University Qingdao China; 5 Department of Endocrinology and Metabolism Peking University Third Hospital Beijing China; 6 Department of Endocrinology and Metabolism Zhongshan Hospital Affiliated to Fudan University Shanghai China; 7 Department of Endocrinology and Metabolism Nanjing First Hospital Nanjing Medical University Nanjing China; 8 Department of Endocrinology Drum Tower Hospital Affiliated to Nanjing University Medical School Nanjing China; 9 Department of Endocrinology The First Medical Center of Chinese People's Liberation Army General Hospital Beijing China

**Keywords:** awareness rate, chronic kidney disease, prevalence, risk factor, screening rate, type 2 diabetes

## Abstract

**Background:**

Chronic kidney disease (CKD) is a significant long-term complication of diabetes and is a primary contributor to end-stage kidney disease.

**Objective:**

This study aimed to report comprehensive nationwide data on the prevalence, screening, and awareness rates of CKD in Chinese patients with type 2 diabetes, along with associated risk factors.

**Methods:**

Baseline data analysis of the ongoing prospective, observational IMPROVE study was conducted. The study cohort comprised patients who had been diagnosed with type 2 diabetes more than 12 months prior, received at least 1 hypoglycemic medication, and were aged ≥18 years. The participants completed questionnaires and underwent laboratory assessments, including blood and urine samples. The data encompassed patient demographics, medical history, concurrent medications, and comorbidities. Comprehensive evaluations involved physical examinations, urinary albumin-to-creatinine ratio (UACR), estimated glomerular filtration rate (eGFR), glycated hemoglobin (HbA_1c_), fasting blood glucose, 2-hour postprandial blood glucose, fasting blood lipid profile, and urinalysis. Descriptive statistics were applied for data interpretation, and logistic regression analyses were used to identify the CKD-associated risk factors in patients with type 2 diabetes.

**Results:**

A national study from December 2021 to September 2022 enlisted 9672 participants with type 2 diabetes from 45 hospitals that had endocrinology departments. The enrollees were from diverse regions in China, as follows: central (n=1221), east (n=3269), south (n=1474), north (n=2219), and west (n=1489). The prevalence, screening, and awareness rates of CKD among patients with type 2 diabetes were 31% (2997/9672), 27% (810/2997), and 54.8% (5295/9672), respectively. Multivariate binary regression analysis revealed that the CKD risk factors were screening, awareness, smoking, age, diabetes duration, concurrent antihypertensive and microcirculation medications, diabetic complications (foot, retinopathy, and neuropathy), hypertension, elevated low-density lipoprotein (LDL) cholesterol, and suboptimal glycemic control. Subgroup analysis highlighted an increased CKD prevalence among older individuals, those with prolonged diabetes durations, and residents of fourth-tier cities. Residents of urban areas that had robust educational and economic development exhibited relatively high awareness and screening rates. Notably, 24.2% (1717/7107) of patients with an eGFR ≥90 mL/min/1.73 m^2^ had proteinuria, whereas 3.4% (234/6909) who had a UACR <30 mg/g presented with an eGFR <60 mL/min/1.73 m^2^. Compared with patients who were cognizant of CKD, those who were unaware of CKD had increased rates of HbA_1c_ ≥7%, total cholesterol >5.18 μmol/L, LDL cholesterol >3.37 μmol/L, BMI ≥30 kg/m^2^, and hypertension.

**Conclusions:**

In a Chinese population of adults with type 2 diabetes, the CKD prevalence was notable, at 31%, coupled with low screening and awareness rates. Multiple risk factors for CKD have been identified.

**Trial Registration:**

ClinicalTrials.gov NCT05047471; https://clinicaltrials.gov/study/NCT05047471

## Introduction

### Background

Diabetes mellitus poses a significant global health challenge, affecting approximately 422 million individuals worldwide, with a majority of patients coming from low- and middle-income regions [1]. This prevalence, especially that of type 2 diabetes, continues to escalate annually because of the surge in risk factors, such as obesity, unhealthy dietary patterns, and sedentary lifestyles. The projections for mainland China estimate a rise in diabetes mellitus cases from 141 million in 2021 to 174 million in 2045 [2]. The morbidity and mortality associated with diabetes predominantly stem from complications that affect vital organ systems, notably the kidneys.

Chronic kidney disease (CKD) has emerged as a prominent long-term complication of diabetes, affecting approximately 30% of patients with type 1 diabetes and 40% of those with type 2 diabetes [3,4]. A cross-sectional study revealed an 8.2% prevalence of CKD among Chinese adults [5]. Globally, CKD is the primary cause of end-stage kidney disease (ESKD), contributing to nearly 40% of new admissions for renal replacement therapy [6]. Among the myriad complications of diabetes, CKD is a pivotal threat and a manifestation of systemic diabetic microangiopathy [7,8]. The escalating prevalence, coupled with the substantial morbidity, mortality, and health care expenditure associated with CKD in patients with diabetes, underscores the need for intensified research endeavors [1].

The rising incidence of diabetes correlates with an increase in ESKD cases [9], with approximately 50% attributed to diabetes-related CKD in high-income nations [10,11]. Notably, the reported prevalences of ESKD in Taiwan and Hong Kong were 43.2% and 46.2%, respectively [12,13]. The economic burden of CKD exerts significant strain on individuals and governments worldwide, affecting health care resource planning and allocation [8,14]. Resources can be optimally used by aligning with patient needs, thereby alleviating costs. Research has indicated that >60% of CKD cases can be detected early through routine screening [15,16]. Timely intervention has been proven to be instrumental in enhancing the quality of life of patients with CKD and curbing the mortality attributable to ESKD [17-19]. The prospective French CKD-REIN cohort study established that achieving at least 2 nephroprotection targets correlated with superior cardiorenal outcomes and reduced the all-cause mortality over a 5-year follow-up period among patients with diabetes and CKD stages 3 and 4 [20]. Nonetheless, despite concerted public health endeavors, CKD awareness among patients remains alarmingly deficient. A systematic review encompassing 32 studies underscored a low CKD awareness rate (19.2%), with the majority of studies reporting that <50% of patients with CKD were cognizant of their condition [21]. A 15-month retrospective analysis in the United States indicated that 51.4%, 52.9%, and 15.2% of patients with type 2 diabetes did not undergo testing for urine protein, urinary albumin-to-creatinine ratio (UACR), and estimated glomerular filtration rate (eGFR), respectively [22]. To mitigate the incidence of diabetes-related CKD, concerted efforts are imperative to increase CKD awareness, implement regular screening for early renal disease, and judiciously administer appropriate medications.

### Objectives

At present, data on the epidemiological profile of CKD among Chinese patients diagnosed with type 2 diabetes are lacking. Furthermore, the levels of awareness and screening remain uncertain. Therefore, the primary objective of this study was to conduct a thorough assessment of the prevailing situation among patients with CKD and type 2 diabetes in China. This assessment encompassed evaluation of the prevalence, awareness levels, screening rates, and identification of the risk factors associated with CKD in this specific population.

## Methods

### Study Design and Participants

Our study involved analysis of baseline data from the ongoing multicenter, observational IMPROVE study. We enrolled both outpatients and inpatients who were diagnosed with type 2 diabetes at 45 hospitals with an endocrinology department across mainland China from December 2021 to September 2022 (Table S1 in Multimedia Appendix 1). Approval to conduct the study and use hospital facilities was obtained from the research ethics committees of all 45 participating centers, and written informed consent was secured from each participant. To safeguard patient privacy, a unique identifier code was assigned and linked with the respective data from each participant.

The participants comprised individuals who were diagnosed with type 2 diabetes at least 12 months prior, undergoing treatment with at least 1 hypoglycemic medication, and aged ≥18 years. Exclusions were made for patients with type 1 diabetes, severe ketosis, diabetic coma, and severe infection or trauma; those who were undergoing dialysis; kidney transplant recipients; and pregnant or lactating women. The participants were asked to answer a questionnaire and underwent laboratory assessments, such as blood and urine sample collection.

The recruitment involved dissemination of materials to the outpatients and inpatients of the endocrinology department. Eligibility was determined based on health status, medical records, and specific criteria, with preference given to individuals who met the inclusion criteria. Subsequently, eligible participants were briefed on the trial procedures, potential risks, and benefits to ensure comprehensive understanding before providing written informed consent.

All study investigators and personnel underwent comprehensive training on the study objectives, tools, and methodologies. A procedural manual and detailed instructions for questionnaire administration were provided. Data entry was facilitated by trained scientific personnel using an electronic data capture system, which enabled real-time patient information updates and facilitated data sharing among the different hospital sites. To ensure data validity and reliability, a stringent quality assurance and control program was implemented. Standard operating procedures for each step were developed and implemented to ensure consistency. The electronic data capture system was used for data collection, storage, transmission, and backup to guarantee data integrity and security. Access to the participant data was restricted to the authorized professional researchers, who regularly monitored the research progress and reviewed the data to promptly identify and rectify any discrepancies.

### Ethical Considerations

The protocol received approval from the research ethics committees of all 45 participating centers (central ethics approval number: S2021-424-01). Each participant provided informed consent. Personal information was deidentified to protect participant confidentiality. No identifying information on the participants was included in any of the tables, figures, and supplementary materials. The participants did not receive compensation for their involvement in the study.

### Clinical and Laboratory Assessment

Patient demographic data, medical history, concomitant drugs, and comorbidities were carefully collected. An extensive assessment was conducted with each patient and comprised a physical examination and laboratory evaluations of UACR; serum creatinine level; eGFR; glycated hemoglobin (HbA_1c_); fasting plasma glucose level; 2-hour postprandial blood glucose; fasting lipid profile, including total cholesterol, triglycerides, high-density lipoprotein cholesterol, and low-density lipoprotein cholesterol (LDL-C); and urine.

All blood and urine samples were analyzed at the central laboratory of each hospital. All the study laboratories successfully completed standardization and certification programs. Blood was collected by venipuncture after an overnight fast of at least 10 hours. Automatic analyzers were used to measure the levels of serum creatinine, HbA_1c_, blood glucose, and lipids and to examine urine samples.

Diabetes-related CKD was defined as the presence of albuminuria or impaired eGFR in individuals with diabetes [23]. The estimation of eGFR used the modified Modification of Diet in Renal Disease (MDRD) equation that was specifically adapted for Chinese patients with CKD [24]. The formula for eGFR (mL/min/1.73 m^2^) was 175 × serum creatinine^–1.234^ × age^−0.179^ × 0.79 (for women). Impaired eGFR was defined as <60 mL/min/1.73 m^2^, and albuminuria was defined as UACR ≥30 mg/g [25,26].

To measure the CKD screening rate, the patients were asked if they had undergone UACR or eGFR testing within the previous year. A response of “yes” indicated that they had undergone CKD screening. To measure the CKD awareness rate, the patients were asked if they knew that they had proteinuria or kidney disease. A response of “yes” and the presence of CKD, according to the definition in this study, indicated that the patients were aware that they had CKD.

### Statistical Analysis

Categorical data are expressed as mean (SD) for continuous data, except UACR, which is expressed as median (IQR) because of the high skewness. The confidence interval was set at 95%, and differences with *P* values <.05 were considered statistically significant. The mean values were compared using 2-tailed unpaired Student *t* tests. The nonparametric Mann-Whitney test was used when a normal distribution was not assumed. Fisher exact tests were used to compare 2 categorical values. The associations between the CKD indicators and relevant covariates were analyzed using logistic regression models. Univariate binary logistic regression analysis was used to identify the potential risk factors for CKD. Subsequently, a multivariate logistic regression analysis was conducted using a forward selection method to determine the independent risk factors. Statistical analyses were performed using SPSS version 28.0 (IBM Corp) and spreadsheet software (Excel, Microsoft Inc).

## Results

### Study Population

A total of 9672 participants with type 2 diabetes (age: mean 57, SD 12.72 years) fulfilled the inclusion criteria. The recruited participants were from different regions in China, including central (n=1221), east (n=3269), south (n=1474), north (n=2219), and west (n=1489). The participating hospitals were categorized as upper first class (32 hospitals), middle first class (11 hospitals), or second class (2 hospitals; Table S1 in Multimedia Appendix 1). The mean duration of diabetes was 9.9 (SD 7.6) years. Compared with participants who had no evidence of kidney damage, those with CKD were older and had a longer disease course; more drug combinations; and higher prevalences of cardiovascular disease, hypertension, diabetic retinopathy, and diabetic neuropathy (Table 1). Of the 2997 participants with CKD, 7.8% (n=234) had a UACR level <30 mg/g, 57.3% (n=1717) had an eGFR ≥90 mL/min/1.73 m^2^, 78.74% (n=2360) had an HbA_1c_ ≥7%, and 66.34% (n=1988) had a fasting glucose level ≥7 mmol/L. More than 60% (1838/2997, 61.3%) of the patients with CKD were overweight or obese. Table S2 in Multimedia Appendix 1 summarizes the demographic characteristics of the population in each region. Notably, 52.1% (1673/2997) of the patients with CKD were newly diagnosed; the remaining patients were previously diagnosed with CKD, and, in most cases, the underlying cause was diabetic nephropathy (data not shown).

**Table 1:** Characteristics of participants with type 2 diabetes and kidney damage indicators: baseline data from the ongoing multicenter observational IMPROVE study in mainland China (December 2021 to September 2022).

Characteristics		Participants with no indicators of kidney damage (n=6675)	Participants with eGFR^a^ <60 mL/min/1.73 m^2^ or UACR^b^ ≥30 mg/g (n=2997)	*P* value	Total sample (n=9672)
Age (years), mean (SD)		56.67 (12.59)	59.35 (12.83)	<.001	57.50 (12.72)
Men, n (%)		3981 (59.6)	1771 (59.1)	.61	5752 (59.5)
Current smoker, n (%)		1423 (21.3)	694 (23.2)	.046	2117 (21.9)
Current drinker, n (%)		976 (14.6)	422 (14.1)	.48	1398 (14.5)
Course of diabetes (years), mean (SD)		9.21 (7.32)	11.34 (8.11)	<.001	9.87 (7.64)
BMI (kg/m^2^), mean (SD)		24.94 (3.79)	25.31 (3.99)	<.001	25.05 (3.85)
Waist-to-hip ratio, mean (SD)		0.92 (0.08)	0.92 (0.08)	.001	0.92 (0.08)
Systolic blood pressure (mm Hg), mean (SD)		130.06 (16.40)	134.37 (18.21)	<.001	131.39 (17.10)
Diastolic blood pressure (mm Hg), mean (SD)		79.69 (10.54)	81.37 (11.19)	<.001	80.21 (10.77)
HbA_1c_^c^ (%), mean (SD)		8.15 (2.61)	8.73 (2.22)	<.001	8.33 (2.51)
Fasting C-peptide (ng/mL), mean (SD)		6.76 (42.14)	5.72 (37.17)	.22	6.44 (40.65)
Fasting insulin (μU/mL), mean (SD)		19.57 (37.52)	25.21 (43.29)	<.001	20.07 (39.21)
FBG^d^ (mmol/L), mean (SD)		7.96 (3.84)	8.58 (3.49)	<.001	8.15 (3.74)
2hBG^e^ (mmol/L), mean (SD)		12.15 (7.00)	13.15 (9.18)	<.001	12.46 (7.76)
UACR^e^ (mg/g), median (IQR)		9.53 (4.04-16.71)	61.08 (35.00-164.25)	<.001	14.60 (6.00-32.00)
eGFR (mL/min/1.73 m^2^), mean (SD)		121.34 (35.67)	101.35 (41.45)	<.001	115.15 (38.67)
Uric acid (μmol/L), mean (SD)		320.99 (92.11)	348.91 (103.22)	.001	329.65 (96.55)
Creatinine (μmol/L), mean (SD)		65.51 (16.72)	75.74 (23.84)	<.001	68.55 (19.67)
Triglyceride (mmol/L), mean (SD)		1.86 (1.83)	2.14 (2.21)	<.001	1.95 (1.96)
Total cholesterol (mmol/L), mean (SD)		4.50 (1.30)	4.63 (1.48)	<.001	4.54 (1.36)
HDL-C^f^ (mmol/L), mean (SD)		1.19 (0.34)	1.16 (0.37)	<.001	1.18 (0.35)
LDL-C^g^ (mmol/L), mean (SD)		2.68 (0.92)	2.66 (1.11)	.47	2.67 (0.99)
Hypertension (≥140/90 mm Hg), n (%)		2138 (32)	1301 (41.2)	<.001	3439 (35.2)
Hypertension (≥130/80 mm Hg), n (%)		4561 (68.3)	2262 (71.9)	<.001	6823 (70)
**Concomitant drugs, n (%)**					
	Antihypertensive drugs	2181 (32.7)	1558 (52)	<.001	3739 (38.7)
	Lipid-lowering drugs	3025 (45.3)	1576 (52.6)	<.001	4601 (47.6)
	Anticoagulants and antiplatelet drugs	1182 (17.7)	676 (22.6)	<.001	1858 (19.2)
	Drugs to improve microcirculation	539 (8.1)	378 (12.6)	<.001	917 (9.5)
	None	2241 (33.6)	651 (21.7)	<.001	2892 (29.9)
**Concomitant disease, n (%)**					
	CVD^h^	1125 (16.9)	697 (23.3)	<.001	1822 (18.8)
	Diabetic foot	57 (0.9)	57 (1.9)	<.001	114 (1.2)
	Diabetic retinopathy	832 (12.5)	771 (25.7)	<.001	1603 (16.6)
	Diabetic neuropathy	2405 (36)	1308 (43.6)	<.001	3713 (38.4)

^a^eGFR: estimated glomerular filtration rate.

^b^UACR: urinary albumin to creatinine ratio.

^c^HbA_1c_: glycated hemoglobin.

^d^FBG: fasting blood glucose.

^e^2hBG: 2-hour postprandial blood glucose.

^f^HDL-C: high-density lipoprotein cholesterol.

^g^LDL-C: low-density lipoprotein cholesterol.

^h^CVD: cardiovascular disease.

### Prevalence, Awareness, and Screening of CKD

Among all patients with type 2 diabetes, the prevalence, awareness, and screening rates for CKD were 31% (2997/9672), 27% (810/2997), and 54.8% (5295/9672), respectively. The subgroup analysis revealed that individuals with higher CKD prevalence rates were >60 years old, had disease durations >20 years, and lived in fourth-tier cities. Those with higher awareness rates were 40 years to 59 years old and men, had an eGFR of 30 mL/min/1.73 m² to 59 mL/min/1.73 m² and a UACR >300 mg/g, and sought care at upper first-class hospitals and teaching hospitals. The subgroup with higher screening rates was <40 years old and resided in first-tier cities (Tables 2-4).

**Table 2:** Prevalence of chronic kidney disease in patients with type 2 diabetes (n=9672): baseline data from the ongoing multicenter observational IMPROVE study in mainland China (December 2021 to September 2022).

Characteristics			Cases, n (%)		Odds ratio (95% CI)		*P* value
Overall sample			2997 (31)		N/A^a^		N/A
**Age (years)**							
	<40 (n=995)	247 (24.8)		Reference		N/A	
	40-59 (n=4180)	1192 (28.5)		1.21 (1.03-1.42)		.02	
	≥60 (n=4497)	1558 (34.7)		1.61 (1.37-1.88)		<.001	
**Sex**							
	Women (n=3920)	1226 (31.3)		Reference		N/A	
	Men (n=5752)	1771 (30.8)		0.98 (0.90-1.07)		.63	
**Kidney function (eGFR^b^ [mL/min/1.73 m^2^])**							
	≥90 (n=7107)	1717 (24.2)		Reference		N/A	
	60-89 (n=1977)	692 (35)		1.69 (1.52-1.88)		<.001	
	30-59 (n=588)	588 (100)		1.00 (1.00***-***1.00)		<.001	
**Albuminuria (UACR^c^ [mg/g])**							
	<30 (n=6909)	234 (3.4)		Reference		N/A	
	30-299 (n=2173)	2173 (100)		1.00 (1.00-1.00)		N/A	
	≥300 (n=590)	590 (100)		1.00 (1.00-1.00)		N/A	
**Duration of diabetes (years)**							
	0-5 (n=3501)	902 (25.8)		Reference		N/A	
	6-10 (n=2424)	665 (27.4)		1.09 (0.97**-**1.22)		.16	
	11-20 (n=2758)	993 (36)		1.62 (1.45-1.81)		<.001	
	>20 (n=989)	437 (44.2)		2.28 (1.97-2.64)		<.001	
**Hospital level**							
	Upper first class (n=6900)	2253 (32.7)		Reference		N/A	
	Middle first class (n=2408)	607 (25.2)		0.70 (0.63-0.77)		<.001	
	Second class (n=364)	137 (37.6)		1.24 (1.00-1.55)		<.001	
**Hospital category**							
	Teaching hospital (n=6398)	2038 (31.9)		Reference		N/A	
	Nonteaching hospital (n=3274)	959 (29.3)		0.89 (0.81-0.97)		.01	
**Region**							
	First-tier city (n=4318)	1302 (30.2)		Reference		N/A	
	Second-tier city (n=2423)	685 (28.3)		0.91 (0.82**-**1.02)		.11	
	Third-tier city (n=1749)	536 (30.7)		1.02 (0.91**-**1.15)		.73	
	Fourth-tier city (n=1182)	474 (40.1)		1.55 (1.36-1.77)		<.001	

^a^N/A: not applicable.

^b^eGFR: estimated glomerular filtration rate.

^c^UACR: urinary albumin-to-creatinine ratio.

**Table 3:** Awareness rates of chronic kidney disease in patients with type 2 diabetes (n=2997): baseline data from the ongoing multicenter observational IMPROVE study in mainland China (December 2021 to September 2022).

Characteristics			Cases, n (%)		Odds ratio (95% CI)		*P* value	
Overall sample			810 (27)		N/A^a^		N/A	
**Age (years)**								
	<40 (n=247)	59 (23.9)		Reference		N/A		
	40-59 (n=1192)	367 (30.8)		1.42 (1.03-1.95)		.04		
	≥60 (n=1558)	384 (24.7)		1.04 (0.76-1.43)		.86		
**Sex**								
	Women (n=1226)	291 (23.7)		Reference		N/A		
	Men (n=1771)	519 (29.3)		1.33 (1.13-1.57)		<.001		
**Kidney function (eGFR^b^ [mL/min/1.73 m^2^])**								
	≥90 (n=1717)	455 (26.5)		Reference		N/A		
	60-89 (n=692)	176 (25.4)		0.95 (0.77-1.16)		.63		
	30-59 (n=588)	179 (30.4)		1.21 (0.99-1.49)		.07		
**Albuminuria (UACR^c^ [mg/g])**								
	<30 (n=234)	45 (19.2)		Reference		N/A		
	30-299 (n=2173)	552 (25.4)		1.43 (1.02-2.01)		.05		
	≥300 (n=590)	213 (36.1)		2.37 (1.65-3.42)		<.001		
**Duration of diabetes (years)**								
	0-5 (n=902)	239 (26.5)		Reference		N/A		
	6-10 (n=665)	188 (28.3)		1.09 (0.87-1.37)		.47		
	11-20 (n=993)	276 (27.8)		1.07 (0.87-1.31)		.56		
	>20 (n=437)	107 (24.5)		0.90 (0.69-1.17)		.47		
**Hospital level**								
	Upper first class (n=2253)	661 (29.3)		Reference		N/A		
	Middle first class (n=607)	116 (19.1)		0.57 (0.46-0.71)		<.001		
	Second class (n=137)	33 (24.1)		0.76 (0.51-1.14)		.22		
**Hospital category**								
	Teaching hospital (n=2038)	636 (31.2)		Reference		N/A		
	Nonteaching hospital (n=959)	174 (18.1)		0.49 (0.40-0.59)		<.001		
**Region**								
	First-tier city (n=1302)	361 (27.7)		Reference		N/A		
	Second-tier city (n=685)	231 (33.7)		1.33 (1.09-1.62)		0.01		
	Third-tier city (n=536)	96 (17.9)		0.57 (0.44-0.73)		<.001		
	Fourth-tier city (n=474)	122 (25.7)		0.90 (0.71-1.15)		0.44		

^a^N/A: not applicable.

^b^eGFR: estimated glomerular filtration rate.

^c^UACR: urinary albumin-to-creatinine ratio.

**Table 4:** Screening rates of chronic kidney disease in patients with type 2 diabetes: baseline data from the ongoing multicenter observational IMPROVE study in mainland China (December 2021 to September 2022).

Characteristics			Cases, n (%)		Odds ratio (95% CI)		*P* value
Overall sample (n=9672)			5295 (54.8)		N/A^a^		N/A
**Age (years)**							
	<40 (n=995)	626 (62.9)		Reference		N/A	
	40-59 (n=4180)	2296 (54.9)		0.72 (0.62-0.83)		<.001	
	≥60 (n=4497)	2373 (52.8)		0.66 (0.57-0.76)		<.001	
**Sex**							
	Women (n=3920)	2118 (54)		Reference		N/A	
	Men (n=5752)	3177 (55.2)		1.05 (0.97-1.14)		.25	
**Kidney function (eGFR^b^ [mL/min/1.73 m^2^])**							
	≥90 (n=7107)	3960 (55.7)		Reference		N/A	
	60-89 (n=1977)	1008 (51)		0.83 (0.75-0.91)		<.001	
	30-59 (n=588)	327 (55.6)		1.00 (0.84-1.18)		.99	
**Albuminuria (UACR^c^ [mg/g])**							
	<30 (n=6909)	3763 (54.5)		Reference		N/A	
	30-299 (n=2173)	1210 (55.7)		1.05 (0.95-1.16)		.33	
	≥300 (n=590)	322 (54.6)		1.00 (0.85-1.19)		.99	
**Duration of diabetes (years)**							
	0-5 (n=3501)	1988 (56.8)		Reference		N/A	
	6-10 (n=2424)	1272 (52.5)		0.84 (0.76-0.93)		<.001	
	11-20 (n=2758)	1484 (53.8)		0.89 (0.80-0.98)		.02	
	>20 (n=989)	551 (55.7)		0.96 (0.83-1.10)		.57	
**Hospital level**							
	Upper first class (n=6900)	3338 (48.4)		Reference		N/A	
	Middle first class (n=2408)	1762 (73.2)		2.91 (2.63-3.22)		<.001	
	Second class (n=364)	195 (53.6)		1.23 (1.00-1.52)		.06	
**Hospital category**							
	Teaching hospital (n=6398)	3534 (55.2)		Reference		N/A	
	Nonteaching hospital (n=3274)	1761 (53.8)		0.94 (0.87-1.03)		.18	
**Region**							
	First-tier city (n=4318)	2841 (65.8)		Reference		N/A	
	Second-tier city (n=2423)	932 (38.5)		0.32 (0.29-0.36)		<.001	
	Third-tier city (n=1749)	1056 (60.4)		0.79 (0.71-0.89)		<.001	
	Fourth-tier city (n=1182)	466 (39.4)		0.34 (0.30-0.39)		<.001	

^a^N/A: not applicable.

^b^eGFR: estimated glomerular filtration rate.

^c^UACR: urinary albumin-to-creatinine ratio.

### Prevalence of the Indicators of Kidney Dysfunction by Disease Stage

Table 5 shows that, of the 7107 participants with an eGFR ≥90 mL/min/1.73 m^2^, 24.2% (1717/7107) had albuminuria. Of the 1977 patients with an eGFR of 60 mL/min/1.73 m^2^ to 89 mL/min/1.73 m^2^, 35% (692/1977) had albuminuria. In the cohort of 6909 patients with an UACR <30 mg/g, 3.4% (234/6909) had an eGFR <60 mL/min/1.73 m^2^ (Table 6).

**Table 5:** Prevalence of kidney function indicators by kidney disease stage in patients with type 2 diabetes (n=9672): baseline data from the ongoing multicenter observational IMPROVE study in mainland China (December 2021 to September 2022).

Kidney function indicators	Kidney disease stage, n (%)				
	1 (eGFR^a^ ≥90 mL/min/1.73 m^2^)	2 (eGFR 60-89 mL/min/1.73 m^2^)	3 (eGFR 30-59 mL/min/1.73 m^2^)	3a (eGFR 45-59 mL/min/1.73 m^2^)	3b (eGFR 30-44 mL/min/1.73 m^2^)
Cases	7107 (71.9)	1977 (20)	588 (6)	418 (4.2)	170 (1.7)
Albuminuria^b^ (n=2763)	1717 (24.2)	692 (35)	354 (60.2)	232 (55.5)	122 (71.8)
eGFR <60 mL/min/1.73 m^2^ and/or albuminuria (n=2997)	1717 (24.2)	692 (35)	588 (100)	418 (100)	170 (100)

^a^eGFR: estimated glomerular filtration rate.

^b^Defined as a urinary albumin-to-creatinine ratio >30 mg/g.

**Table 6:** Prevalence of kidney function indicators by albuminuria stage in patients with type 2 diabetes (n=9672): baseline data from the ongoing multicenter observational IMPROVE study in mainland China (December 2021 to September 2022).

Kidney function indicators	Albuminuria^a^ stage, n (%)		
	Normal albuminuria (UACR^b^ >30 mg/g)	Microalbuminuria (UACR 30-299 mg/g)	Massive albuminuria (UACR ≥300 mg/g)
Cases	6909 (69.9)	2173 (22)	590 (6)
eGFR^c^ <60 mL/min/1.73 m^2^ (n=588)	234 (3.4)	195 (9)	159 (27)
eGFR <60 mL/min/1.73 m^2^ and/or albuminuria (n=2997)	234 (3.4)	2173 (100)	590 (100)

^a^Defined as a urinary albumin-to-creatinine ratio >30 mg/g.

^b^UACR: urinary albumin-to-creatinine ratio.

^c^eGFR: estimated glomerular filtration rate.

### Metabolic Indicators and Comorbidities, According to CKD Awareness

In this study, metabolic indicators were compared by level of CKD awareness. Among patients with type 2 diabetes, the proportions of patients with HbA_1c_ ≥7%, total cholesterol >5.18 μmol/L, LDL-C >3.37 μmol/L, BMI ≥30 kg/m^2^, or hypertension were higher in those who were unaware of CKD than in those who were aware of CKD. However, only the difference for patients with HbA_1c_ ≥7% showed statistical significance (Table 7).

**Table 7:** Metabolic indicators and comorbidities of patients with type 2 diabetes, according to their awareness of chronic kidney disease (CKD): baseline data from the ongoing multicenter observational IMPROVE study in mainland China (December 2021 to September 2022).

Metabolic indicators	Cases aware of CKD (n=810), n (%; 95% CI)	Cases unaware of CKD (n=2187), n (%; 95% CI)	*P* value
HbA_1c_^a^ ≥7%	599 (74; 70.44-77.47)	1761 (80.5; 78.67-82.37)	<.001
TC^b^ >5.18 mmol/L	237 (26.3; 23.47-35.05)	675 (30.9; 27.38-34.35)	.39
TG^c^ >1.7 (mmol/L)	360 (44.4; 39.31-49.58)	906 (41.4; 38.22-44.63)	.14
LDL-C^d^ >3.37 (mmol/L)	187 (23.1; 17.05-29.13)	533 (24.4; 20.73-28.02)	.46
BMI ≥25 kg/m^2^	373 (46.1; 40.99-51.11)	1051 (48.1; 45.04-51.08)	.33
BMI ≥30 kg/m^2^	76 (9.4; 2.83-15.94)	230 (10.5; 6.55-14.48)	.35
Hypertension (≥130/80 mm Hg)	605 (74.7; 71.23-78.16)	1657 (75.8; 73.70-77.83)	.55

^a^HbA_1c_: glycated hemoglobin.

^b^TC: total cholesterol.

^c^TG: triglyceride.

^d^LDL-C: low-density lipoprotein cholesterol.

### Risk Factors for CKD in Patients With Type 2 Diabetes

The factors associated with CKD in the univariate analyses included awareness; age; duration of diabetes; concurrent use of antihypertensive drugs, lipid regulators, antiplatelet agents, and microcirculation-improving agents; absence of concomitant drug use, except hypoglycemic agents; BMI; hypertension; elevated LDL-C and poor glycemic control; and diabetic foot, diabetic retinopathy, diabetic neuropathy, diabetic lower extremity arteriopathy, and cardiovascular disease. The subsequent multivariate binary regression analysis indicated that screening and awareness were significantly associated with CKD. Age and duration of diabetes were identified as risk factors for CKD. Among the concurrent medications, only antihypertensive and microcirculation-improving drugs were associated with CKD. Moreover, diabetic complications, such as diabetic foot, diabetic retinopathy, cerebrovascular disease, and hypertension, were risk factors for CKD. Poor lipid and glucose control also contributed to the risk of CKD (Table 8).

**Table 8:** Risk factors for chronic kidney disease (CKD) in patients with type 2 diabetes: baseline data from the ongoing multicenter observational IMPROVE study in mainland China (December 2021 to September 2022).

Item		Univariate regression			Multivariate regression	
		OR^a^ (95% CI)	*P* value	OR (95% CI)		*P* value
Screening for CKD		1.012 (0.928-1.104)	.78	0.791 (0.715-0.875)		<.001
Aware of CKD		2.542 (2.281-2.832)	<.001	2.720 (2.401-3.081)		<.001
Sex		0.978 (0.895-1.067)	.61	—^b^		—
**Smoking**						
	Never (reference)	1.000	N/A^c^	1.000		N/A
	Ever	0.957 (0.852-1.075)	.46	0.847 (0.747-0.960)		.009
	Current	1.101 (0.990-1.225)	.08	1.105 (0.986-1.239)		.09
**Drinking**						
	Never (reference)	1.000	N/A	—		—
	Ever	0.920 (0.820-1.032)	.15	—		—
	Current	0.940 (0.829-1.066)	.34	—		—
**Age (years)**						
	18-40 (reference)	1.000	N/A	1.000		N/A
	41-65	1.236 (1.067-)1.431	.005	1.072 (0.915-1.256)		.39
	>65	1.838 (1.572-2.1500	<.001	1.401 (1.174-1.671)		<.001
**Duration of diabetes (years)**						
	0-5 (reference)	1.000	N/A	1.000		N/A
	6-10	1.089 (0.969-1.225)	.15	0.968 (0.855-1.097)		.61
	11-20	1.621 (1.454-1.807)	<.001	1.241 (1.101-1.399)		<.001
	>20	2.281 (1.970-2.641)	<.001	1.603 (1.360-1.891)		<.001
Family history of diabetes		1.007 (0.914-1.109)	.89	—		—
Family history of CKD		1.097 (0.801-1.503)	.56	—		—
Use of antihypertensive drugs		2.231 (2.043-2.436)	<.001	1.751 (1.590-1.928)		<.001
Use of lipid regulators		1.338 (1.227-1.459)	<.001	—		—
Use of anticoagulants		1.506 (0.966-2.346)	.07	—		—
Use of antiplatelet agents		1.355 (1.217-1.509)	<.001	—		—
Use of drugs to improve microcirculation		1.643 (1.430-1.888)	<.001	1.276 (1.094-1.488)		.002
No concomitant drug use		0.549 (0.497-0.607)	<.001	—		—
Diabetic foot		2.251 (1.555-3.258)	<.001	2.269 (1.534-3.357)		<.001
Diabetic retinopathy		2.432 (2.180-2.714)	<.001	1.887 (1.669-2.133)		<.001
Diabetic neuropathy		1.375 (1.259-1.501)	<.001	0.882 (0.793-0.980)		.02
Diabetic lower extremity vascular disease		1.268 (1.128-1.425)	<.001	0.865 (0.757-0.988)		.03
Cardiovascular and cerebrovascular diseases		1.495 (1.344-1.663)	<.001	—		—
Cerebrovascular disease		1.814 (1.513-2.174)	<.001	1.282 (1.052-1.562)		.01
Coronary heart disease		1.362 (1.185-1.564)	<.001	—		—
CVD^d^ (narrow)		1.366 (1.157-1.612)	<.001	—		—
**BMI (kg/m^2^)**						
	<18.5 (reference）	1.000	N/A	—		—
	18.5-24.9	0.906 (0.656-1.251)	.55	—		—
	25-29.9	0.995 (0.719-1.377)	.98	—		—
	30-34.9	1.198 (0.843-1.704)	.31	—		—
	35-39.9	1.102 (0.660-1.839)	.71	—		—
	≥40	1.111 (0.541-2.281)	.77	—		—
Hypertension (≥140/90 mm Hg)		1.631 (1.493-1.782)	<.001	1.368 (1.244-1.505)		<.001
Elevated LDL-C^e^ (>3.37 mmol/L)		1.227 (1.107-1.359)	<.001	1.219 (1.091-1.362)		<.001
Poor glycemic control (HbA_1c_^f^ ≥7%)		2.056 (1.859-2.274)	<.001	1.810 (1.624-2.017)		<.001

^a^OR: odds ratio.

^b^Not included in the model.

^c^N/A: not applicable.

^d^CVD: cardiovascular disease.

^e^LDL-C: low-density lipoprotein cholesterol.

^f^HbA_1c_: glycated hemoglobin.

### Treatment Status of Patients With Type 2 Diabetes

Among patients with type 2 diabetes and CKD, insulin, metformin, and sodium-glucose cotransporter 2 inhibitors were the 3 most commonly prescribed hypoglycemic medications, with utilization rates exceeding 40% (Table S3 in Multimedia Appendix 1). Sodium-glucose cotransporter 2 inhibitors were more frequently prescribed in the CKD group than in the non-CKD group. However, metformin use was higher in the non-CKD group than in the CKD group (Figure S1 and Table S4 in Multimedia Appendix 1).

## Discussion

### Principal Findings

This investigation involved a large number of outpatients and inpatients with type 2 diabetes from 45 hospitals in China. The prevalence, screening, and awareness rates in this population were 31%, 54.8%, and 27%, respectively. Patients who were aware of CKD had better glycemic control and metabolic indicators. Our study identified multiple risk factors, such as age, duration of diabetes, and poor glycemic control, as risk factors for CKD. A major strength of our study was the use of a nationwide representative survey involving multiple provinces and cities across China in recent years, thereby providing a unique data set that was used to explore the diagnosis and treatment status of CKD. Furthermore, training programs and quality control procedures ensured the credibility of the results.

The incidence of diabetes-related CKD in the general population is 1.23% [27], accounting for a significant proportion of patients with diabetes. CKD reportedly occurs in approximately 30% to 40% of patients with type 2 diabetes in Hong Kong and Japan [28-30]. A study from the United States reported that the prevalence of elevated albuminuria is 15% [31]. To date, large-scale studies on the CKD prevalence in China are lacking. A meta-analysis found that the pooled prevalence of CKD in patients with type 2 diabetes in China, as a whole, was 21.8% [32]. Our nationwide survey revealed that the prevalence of CKD in patients with type 2 diabetes was 31%. With the increasing prevalence of type 2 diabetes in our country, the prevalence of CKD may increase. According to our subgroup analysis, patients >60 years old with type 2 diabetes had the highest prevalence of CKD (34.7%). The demographic transition to an aging population likely contributed to the relatively high prevalence of both type 2 diabetes and coexisting CKD in this subgroup [33].

Despite its high prevalence and significant health care costs for affected individuals, CKD awareness and screening rates remain notably low [34]. A survey on CKD and cardiovascular risk across 12 countries found that only 6% of the general population were aware of their CKD status, and 31% of patients with CKD were unaware of their condition [35]. Similarly, a study involving 176,874 adults from 31 provinces in China reported a CKD awareness rate of 10% [5]. Meanwhile, nearly one-half of patients with type 2 diabetes in the United States were reported to have not undergone annual eGFR and UACR testing, despite recommendations from the American Diabetes Association [31]. Our study indicated that only 27% of patients were aware of their CKD status, with an overall CKD screening rate of 54.8% among patients with type 2 diabetes. These findings suggest a lack of attention to CKD among Chinese patients with diabetes, as well as physicians. Furthermore, the better control of metabolic indicators in informed patients with CKD than in uninformed patients highlights the importance of raising public awareness of CKD. Moreover, the relatively high screening and awareness rates in teaching hospitals and economically developed areas in this study indicate the potential impact of hospital management and education on improving patient awareness and screening rates [36,37].

In this study, 24.2% of individuals with an eGFR ≥90 mL/min/1.73 m^2^ had albuminuria. Both UACR and eGFR are measures of glomerular function. Albuminuria mainly reflects glomerular capillary wall permeability to macromolecules; detectable increases in albuminuria generally occur before eGFR decline, with early changes possibly indicating signs of kidney disease [38-40]. Similarly, proteinuria is associated with an increased risk of hospitalization and cardiovascular events in older adults, further emphasizing the importance of proteinuria measurements [41-43]. In addition to its utility for early detection of CKD in individuals with type 2 diabetes, the UACR can be used to monitor the progression of CKD [44]. eGFR alone is suitable for testing at CKD stage ≥3 but not in the early stages of CKD, during which the eGFR may not be sufficiently impaired and other markers of kidney damage are required for detection. As an early marker of CKD, the UACR has been recommended for all patients with diabetes [45]. Studies have shown that, compared with eGFR testing, UACR testing was underused [22,46,47] and, therefore, needs greater attention. However, it should also be noted that some patients with type 2 diabetes may have CKD without albuminuria [48,49]. Our study found that 3.3% of patients with normal albuminuria had an eGFR <60 mL/min/1.73 m^2^. The American Diabetes Association recommends annual testing for CKD using eGFR and UACR [31,50,51]. When used together, eGFR and UACR were shown to improve risk stratification and diagnostic accuracy [51,52].

Although the prevalence of type 2 diabetes is increasing in China, the exact risk factors for CKD remain unclear. In this study, the awareness and screening rates for CKD were associated with the risk of developing CKD. The prevalence of CKD was low in populations with high screening rates. Therefore, improving the screening and identification of CKD is essential to reduce the prevalence and burden of the disease in patients with type 2 diabetes. A cost-effectiveness analysis revealed that optimal strategies for CKD screening led to improvements in patient outcomes, including reduced cumulative incidence of ESKD, improved life expectancy, and reduced mean costs [53]. This study found that hypertension, cerebrovascular disease, and concomitant use of antihypertensive or microcirculation-improving drugs were associated with the risk of developing CKD. Many studies have demonstrated a relationship between high blood pressure and albuminuria [54] and the beneficial renoprotective effects of angiotensin-converting enzyme inhibitors and angiotensin 2 receptor blockers on the progression of CKD in patients with hypertension and diabetes [55-59]. The rational use of antihypertensive or microcirculation-improving drugs can help delay the development of CKD in patients with diabetes.

### Limitations

Several limitations were observed in this study. First, the cross-sectional design restricted our ability to establish causal relationships between CKD and its associated factors. The regression analysis results might have been influenced by unaccounted variables. Second, our study solely relied on eGFR and albuminuria to define CKD, because data on the other markers of kidney damage were lacking. Consequently, the prevalence of CKD might have been underestimated because of the absence of information on cases with an eGFR <30 mL/min/1.73 m^2^. Third, our study focused exclusively on urban populations and possibly neglected the potential differences in CKD prevalence between rural and urban settings. Fourth, the study was confined to patients who were treated in endocrinology departments and potentially overlooked CKD cases that were seen at the other departments; this might have affected the prevalence estimations. Fifth, the inclusion criterion of a diabetes duration >12 months potentially overestimated the prevalence of CKD, which is often associated with a longer diabetes duration. Last, the analysis of risk factors remained incomplete, with limited exploration of lifestyle and environmental influences.

### Conclusions

This cross-sectional study underscored a concerning 31% prevalence of CKD and notably low rates of CKD screening and awareness among adult Chinese patients with type 2 diabetes. Notably, the UACR was highlighted as a valuable tool for early CKD detection. Encouragingly, patients who were cognizant of their CKD status demonstrated improved management of blood glucose levels and metabolic parameters. However, overall disease control among individuals with CKD and type 2 diabetes in China remains inadequate. Our results on age, diabetes duration, and suboptimal glycemic control as the significant contributors to CKD development highlight the need to enhance screening efforts, strengthen awareness campaigns, and meticulously identify and manage risk factors to effectively mitigate the CKD burden in this population. 

## Appendix

Multimedia Appendix 1 Supplementary material.

## Abbreviations

CKD: chronic kidney disease
eGFR: estimated glomerular filtration rate
ESKD: end-stage kidney disease
HbA1c: glycated hemoglobin
LDL-C: low-density lipoprotein cholesterol
MDRD: Modification of Diet in Renal Disease
UACR: urinary albumin-to-creatinine ratio

## Data Availability

The data sets used and/or analyzed during the current study are available from the corresponding author on reasonable request.
