# Supplementary material for: Prevalence and Risk Factors of Chronic Kidney Disease in Patients With Type 2 Diabetes in China: Cross-Sectional Study
Source: JMIR Public Health Surveill. 2024 Aug 30;10:e54429. doi: 10.2196/54429 (PMC11399742; doi:10.2196/54429)
Supplement: Multimedia Appendix 1 [file publichealth_v10i1e54429_app1.pdf]

Prevalence and Risk Factors of Chronic Kidney Disease in Patients with Type 2 Diabetes in China: Cross-Sectional Study

Supplementary material

Supplementary Figure S1. Situation of the use of hypoglycemic medication in patients with type 2 diabetes in China. DPP-4 inhibitors, Dipeptidyl peptidase IV inhibitors; GLP-1 receptor agonist, Glucagon-like peptide-1 receptor agonist; SGLT-2 inhibitors, Sodium-glucose cotransporter-2 inhibitors.

Supplementary Table S1. List of 45 participating centers.

Supplementary Table S2. Demographic characteristics of the population participating in the study in different regions.

Supplementary Table S3. Treatment status of patients with type 2 diabetes in China.

Supplementary Table S4. The detailed information of the use of hypoglycemic medication in patients with type 2 diabetes in China.

Supplementary Figure S1. Situation of the use of hypoglycemic medication in patients with type 2 diabetes in China. DPP-4 inhibitors, Dipeptidyl peptidase IV inhibitors; GLP-1 receptor agonist, Glucagon-like peptide-1 receptor agonist; SGLT-2 inhibitors, Sodium-glucose cotransporter-2 inhibitors.

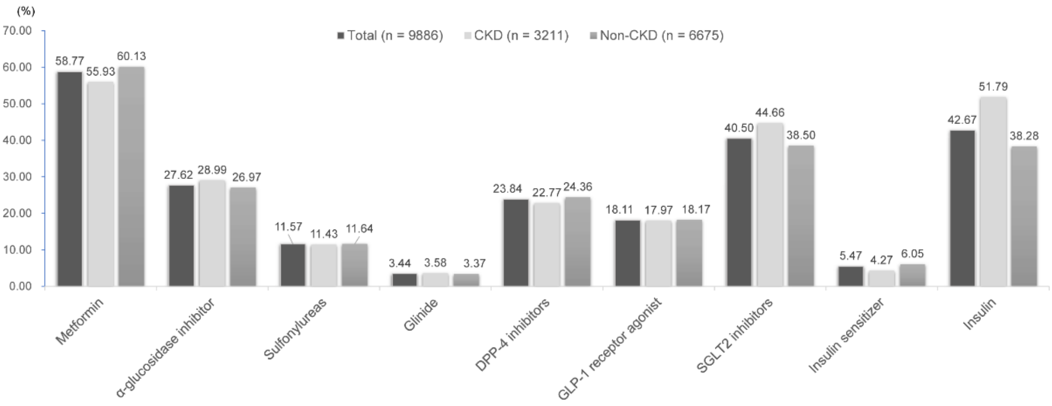

Supplementary Table S1. List of 45 participating centers.

Number	Hospital name	Level	Province	Region
1	Tongji Hospital, Tongji Medical College Of HUST	upper first-class	Hubei	Central
2	Wuhan No. 4 Hospital	upper first-class	Hubei	Central
3	The Central Hospital of Wuhan	upper first-class	Hubei	Central
4	Renmin Hospital of Wuhan University	upper first-class	Hubei	Central
5	Wuhan No. 1 Hospital	upper first-class	Hubei	Central
6	Wuhan No. 3 Hospital	upper first-class	Hubei	Central
7	Nanjing Drum Tower Hospital (The Affiliated Hospital of Nanjing University Medical School)	upper first-class	Jiangsu	East
8	Nantong First People's Hospital	middle first-class	Jiangsu	East
9	Zhenjiang First People's Hospital	middle first-class	Jiangsu	East
10	The Affiliated Hospital of Xuzhou Medical University	middle first-class	Jiangsu	East
11	The First Affiliated Hospital of Soochow University	upper first-class	Jiangsu	East
12	Yixing People's Hospital	middle first-class	Jiangsu	East
13	Taizhou Second People's Hospital	middle first-class	Jiangsu	East
14	Suzhou Ninth People's Hospital	upper first-class	Jiangsu	East
15	The Second Affiliated Hospital of Soochow University	upper first-class	Jiangsu	East
16	The Affiliated Hospital of Qingdao University	upper first-class	Shandong	East
17	Qingdao Third People's Hospital	middle first-class	Shandong	East
18	Weihai Municipal Hospital	upper first-class	Shandong	East
19	People's Hospital of Rizhao	upper first-class	Shandong	East
20	Qingdao Eighth People's Hospital	middle first-class	Shandong	East
21	Zhongshan Hospital affiliated to Fudan University	upper first-class	Shanghai	East
22	Shanghai Punan Hospital of Pudong New District	second-class	Shanghai	East
23	Shanghai Seventh People's Hospital	upper first-class	Shanghai	East
24	Wusong Hospital, Zhongshan Hospital affiliated to Fudan University	second-class	Shanghai	East
25	Ningbo First Hospital	upper first-class	Zhejiang	East
26	The First Medical Center of Chinese PLA General Hospital	upper first-class	Beijing	North
27	Beijing Bo'ai Hospital	upper first-class	Beijing	North
28	The First Affiliated Hospital of Tsinghua University	middle first-class	Beijing	North
29	Peking University Shougang Hospital	middle first-class	Beijing	North
30	Beijing Friendship Hospital， Capital Medical University	upper first-class	Beijing	North
31	The 6th Medical Center of Chinese PLA General Hospital	upper first-class	Beijing	North

(continued)

Supplementary Table S1. *Continued.*

Number	Hospital name	Level	Province	Region
32	Peking University Third Hospital	upper first-class	Beijing	North
33	Peking University Third Hospital Yanqing Hospital	middle first-class	Beijing	North
34	Beijing Haidian Hospital	upper first-class	Beijing	North
35	Beijing Luhe Hospital, Capital Medical University	middle first-class	Beijing	North
36	Nanfang Hospital, Southern Medical University	upper first-class	Guangdong	South
37	Shenzhen Second People's Hospital	upper first-class	Guangdong	South
38	Sun Yat-sen Memorial Hospital, Sun Yat-sen University	upper first-class	Guangdong	South
39	Guangzhou First People's Hospital	upper first-class	Guangdong	South
40	Guangdong Provincial People's Hospital	upper first-class	Guangdong	South
41	The Affiliated Hospital of Guizhou Medical University	upper first-class	Guizhou	West
42	The Second Affiliated Hospital of Guizhou Medical University	upper first-class	Guizhou	West
43	Guiyang First People's Hospital	upper first-class	Guizhou	West
44	The Affiliated Hospital of Zunyi Medical University	upper first-class	Guizhou	West
45	Zunyi First People's Hospital	upper first-class	Guizhou	West

Supplementary Table S2. Demographic characteristics of the population participating in the study in different regions.

**Region**		**Central**	**East**	**South**	**North**	**West**	**Total**
n	n (%)	1221 (12.62%)	3269 (33.80%)	1474 (15.24%)	2219 (22.94%)	1489 (15.39%)	9672 (100%)
Age (year)	mean (sd)	59.33 (11.11)	57.71 (13.12)	55.04 (12.08)	56.51 (13.64)	59.43 (11.70)	57.50 (12.72)
Course of diabetes (year)	mean (sd)	10.62 (7.25)	10.08 (7.91)	9.16 (6.98)	9.74 (8.06)	9.66 (7.24)	9.87 (7.64)
Men	n (%)	694 (56.84%)	1964 (60.08%)	891 (60.45%)	1306 (58.86%)	897 (60.24%)	5752 (59.47%)
Current smoker	n (%)	768 (62.90%)	2080 (63.63%)	817 (55.43%)	1287 (58.00%)	1032 (69.31%)	5984 (61.87%)
Current drinker	n (%)	444 (36.36%)	1702 (52.06%)	569 (38.60%)	1059 (47.72%)	788 (52.92%)	4562 (47.17%)
BMI ≥ 25	n (%)	495 (40.54%)	1440 (44.05%)	579 (39.28%)	1197 (53.94%)	679 (45.60%)	4390 (45.39%)
Waist-to-Hip Ratio, WHR (Female > 0.7, Male > 0.9)	n (%)	862 (70.60%)	2495 (76.32%)	1288 (87.38%)	1643 (74.04%)	1205 (80.93%)	7493 (77.47%)
Hypertension(140/90mmHg)	n (%)	455 (37.26%)	1325 (40.53%)	394 (26.73%)	685 (30.87%)	580 (38.95%)	3439 (35.56%)
eGFR < 60 mL/min per 1·73 m² and/or UACR ≥ 30 mg/g	n (%)	444 (36.36%)	987 (30.19%)	545 (36.97%)	543 (24.47%)	478 (32.10%)	2997 (30.99%)
HbA1c ≥ 7%	n (%)	846 (69.29%)	2249 (68.80%)	931 (63.16%)	1488 (67.06%)	1139 (76.49%)	6653 (68.79%)
TC > 5.18(mmol/L)	n (%)	289 (23.67%)	852 (26.06%)	491 (33.31%)	518 (23.34%)	423 (28.41%)	2573 (26.60%)
TG > 1.7 (mmol/L)	n (%)	502 (41.11%)	1055 (32.27%)	499 (33.85%)	779 (35.11%)	738 (49.56%)	3573 (36.94%)
LDL-C > 3.37(mmol/L)	n (%)	228 (18.67%)	568 (17.38%)	475 (32.23%)	492 (22.17%)	325 (21.83%)	2088 (21.59%)

Supplementary Table S3. Treatment status of patients with type 2 diabetes in China.

Indicators	No. of Cases/Participants (n=2997)	% (95% CI)
**eGFR (mL/min per 1.73 m^2)**		
≥ 90	1717/2997	57.29% (54.95%-59.63%)
60-89	692/2997	23.09% (19.95%-26.23%)
30-59	588/2997	19.62% (16.41%-22.83%)
**UACR (mg/g)**		
< 30	234/2997	7.81% (4.37%-11.25%)
30-299	2194/2997	73.21% (71.35%-75.06%)
≥ 300	569/2997	18.99% (15.76%-22.21%)
**Course of diabetes (years)**		
0-5	653/2997	21.79% (18.62%-24.95%)
6-10	674/2997	22.49% (19.34%-25.64%)
11-20	1064/2997	35.50% (32.63%-38.38%)
> 20	606/2997	20.22% (17.02%-23.42%)
**FPG (mmol/l)**		
< 7	1009/2997	33.67% (30.75%-36.58%)
≥ 7	1988/2997	66.33% (64.26%-68.41%)
**HbA1c (%)**		
< 7	637/2997	21.25% (18.08%-24.43%)
≥ 7	2360/2997	78.75% (77.09%-80.40%)
**BMI (kg/m^2)**		
< 18.5	56/2997	1.87% (0.31%-5.42%)
18.5-24	1103/2997	36.80% (33.96%-39.65%)
24-28	1216/2997	40.57% (37.81%-43.33%)
≥ 28	622/2997	20.75% (17.57%-23.94%)

FBG, fasting blood glucose; UACR, urinary albumin to creatinine ratio.

Supplementary Table S4. The detailed information of the use of hypoglycemic medication in patients with type 2 diabetes in China.

**Hypoglycemic medication**	**Total (n=9672)**		**Participants with no Indicators of kidney damage (n=6675)**		**Participants with eGFR < 60 mL/min per 1.73 m² and/or UACR ≥ 30 mg/g (n=2997)**	
	**No. of Cases/Participants**	**% (95% CI)**	**No. of Cases/Participants**	**% (95% CI)**	**No. of Cases/Participants**	**% (95% CI)**
Metformin	5692/9672	58.85% (57.57% ~60.13%)	4014/6675	60.13% (58.62%-61.65%)	1678/2997	55.99% (53.61%-58.36%)
a-glucosidase inhibitor	2688/9672	27.79% (26.10% ~29.49%)	1800/6675	26.97% (24.92%-29.02%)	889/2997	29.66% (26.66%-32.67%)
SU	1115/9672	11.53% (9.65% ~13.40%)	777/6675	11.64% (9.39%-13.90%)	338/2997	11.28% (7.91%-14.65%)
GLN	328/9672	3.39% (1.43% ~5.35%)	225/6675	3.37% (1.01%-5.73%)	103/2997	3.44% (0.10%-6.95%)
DPP-4 inhibitors	2304/9672	23.82% (22.08% ~25.56%)	1626/6675	24.36% (22.27%-26.45%)	678/2997	22.62% (19.47%-25.77%)
GLP-1 receptor agonist	1769/9672	18.29% (16.49% ~20.09%)	1213/6675	18.17% (16.00%-20.34%)	556/2997	18.55% (15.32%-21.78%)
SGLT2 inhibitors	3970/9672	41.05% (39.52% ~42.58%)	2570/6675	38.50% (36.62%-40.38%)	1400/2997	46.71% (44.10%-49.33%)
Insulin sensitizer	536/9672	5.54% (3.60% ~7.48%)	404/6675	6.05% (3.73%-8.38%)	132/2997	4.40% (0.90%-7.90%)
Insulin	4124/9672	42.64% (41.13% ~44.15%)	2555/6675	38.28% (36.39%-40.16%)	1569/2997	52.35% (49.88%-54.82%)

DPP-4 inhibitors, dipeptidyl peptidase IV inhibitors；GLN, glinides；GLP-1 receptor agonist, glucagon-like peptide-1 receptor agonist; SGLT-2 inhibitors, sodium-glucose cotransporter-2 inhibitors; SU, sulfonylurea; UACR, urinary albumin to creatinine ratio.
